# *Salmonella enterica* serovar Virchow meningitis in a young man in Italy: a case report

**DOI:** 10.1186/1752-1947-8-139

**Published:** 2014-05-06

**Authors:** Daniela Lombardi, Silvana Malaspina, Angela Strippoli, Claudia Lucarelli, Ida Luzzi, Giancarlo Ripabelli

**Affiliations:** 1 Regional Reference Service of Epidemiology for the Surveillance, Prevention and Control of Infectious Diseases SeREMI ASL AL, Alessandria, Italy; 2 Infectious Diseases of Hygiene Service and Public Health of ASL TO1, Turin, Italy; 3 Department of Infectious, Parasitic and Immuno-Mediated Diseases, Istituto Superiore di Sanità, Rome, Italy; 4 Department of Medicine and of Health Sciences, University of Molise, Campobasso, Italy; 5 Coordinator of EnterNet Italia Surveillance Network for foodborne and waterborne diseases, Istituto Superiore di Sanità, Viale Regina Elena, 299, 00161 Roma, Italy; 6 European Public Health Microbiology Training Programme (EUPHEM), European Centre for Disease Prevention and Control (ECDC), Tomtebodavägen 11a, 171 83, Stockholm, Sweden

**Keywords:** Carrier state, Gastroenteritis, Meningitis, *Salmonella* Virchow

## Abstract

**Introduction:**

*Salmonella enterica* is a leading cause of foodborne infections worldwide and includes more than 2500 different serovars, causing primarily gastroenteritis. However, the infection may occur elsewhere and produce characteristic clinical syndromes. Meningitis is a rare complication that occurs in less than 1% of clinical salmonellosis.

**Case presentation:**

We describe a case of *Salmonella* Virchow meningitis in a 36-year-old Caucasian man presenting with headache in the occipital region, associated fever, nausea and vomiting, dyspnea and ambulatory difficulty. The cerebrospinal fluid culture showed growth of *Salmonella*, later confirmed to be *Salmonella enterica* serovar Virchow.

**Conclusions:**

*Salmonella* Virchow infection is rare and this report highlights the risk of meningitis as a presentation of salmonellosis. To the best of our knowledge this is the first Italian case of meningitis due to *Salmonella* Virchow in a young adult. The probable route of transmission remains unclear and a long carriage state after a previous episode of gastroenteritis should be considered.

## Introduction

*Salmonella enterica* represents a leading cause of foodborne infections worldwide and includes more than 2500 different serovars, which primarily cause gastroenteritis. However, infection at other sites may occur, producing characteristic clinical syndromes. Meningitis is a rare complication which is diagnosed in less than 1% of clinical salmonellosis [1]. In Italy, dedicated surveillance systems for invasive bacterial diseases [2] and for human gastrointestinal infections (ENTER-NET Italia) [3] have been active since 1994 and 1980, respectively. The two networks are coordinated by the Istituto Superiore di Sanità (ISS), Rome, Italy, in collaboration with the Regional Health Institutions.

In this paper, we describe the first Italian case of *Salmonella* Virchow meningitis in an adult. Meningitis was eventually related to a long carriage state after a previous episode of gastroenteritis and the case was detected by the two dedicated Italian surveillance systems.

## Case presentation

A 36-year-old Caucasian man was admitted to hospital in November 2010 due to fever (38.5°C) associated with chills, occipital headache, nausea and vomiting but not diarrhea, neck pain, asthenia, dyspnea, slight neck stiffness and ambulatory difficulty. His cerebrospinal fluid (CSF) showed an elevated protein level (588mg/dL), glucose 31mg/dL and a white blood cell count of 3500/μL. On the basis of the clinical observation and laboratory findings, a provisional diagnosis of bacterial meningitis was made and empiric therapy of intravenous ceftriaxone and sulbactam/ampicillin was started before he was transferred to a different hospital specialized for infectious diseases.

He reported meningococcal ACWY vaccination in 2003 during his stay in Senegal, Africa. Hence, a clinical evaluation and microbiological investigations for *Neisseria meningitidis* and *Haemophilus influenzae* were performed. During the night his health condition improved, probably due to the antimicrobial therapy. On the following day, a diagnostic nested-polymerase chain reaction was performed on deoxyribonucleic acid (DNA) extracted from his CSF specimen [4], which excluded infection by *N. meningitidis*, *H. influenzae*, *Streptococcus pneumoniae* and *Streptococcus agalactiae*. In the meantime, the CSF culture showed growth of *Salmonella*, later confirmed to be *Salmonella enterica* serovar Virchow by the ISS reference laboratory. Culture from feces or blood was not performed because he was under treatment with antimicrobials.

An antimicrobial susceptibility test that was performed following guidelines by the National Committee for Clinical Laboratory Standards showed that the strain was susceptible to all antibiotics tested, (nalidixic acid, ampicillin, cefotaxime, chloramphenicol, gentamicin, kanamycin, streptomycin, sulphonamides, tetracycline, and trimethoprim–sulphamethoxazole) but showed a reduced susceptibility to ciprofloxacin (minimum inhibitory concentration >0.25μg/mL).

Past patient history revealed that in September 2010 he attended a wedding party in Spain where he ate raw fish. After 48 hours he developed gastroenteritis together with two other guests. He did not seek medical examination, and underwent self-treatment with antidiarrheal drugs and probiotics; no microbiological or epidemiological data are available. At the end of September he returned to Italy where he was admitted to a hospital emergency unit for the occurrence of an itchy papule, reporting a previous bronchopulmonary infection which was treated with ciprofloxacin and levofloxacin for 25 days. After 4 days he presented again to the hospital because of a persistent fever; pneumonia in the resolution phase was diagnosed and therapy with macrolides was started.

Antimicrobial intravenous therapy was continued (ampicillin/sulbactam and ceftriaxone) together with administration of anti-edema medications and steroids. The course was regular without any complication: he remained afebrile and his headache resolved slowly.

He was discharged home after 15 days in good condition. He was recommended to undergo additional tests to assess his immunologic status due to previous diagnosed deficit of CD4+ T lymphocytes and immunoglobulin M positivity for *Cytomegalovirus* but he declined to have further investigation.

## Discussion

*Salmonella enterica* serovar Virchow belongs to Group C and is rarely isolated from gastrointestinal infection, accounting for 0.1% to 0.5% in Italy and 0.8% in the European Union of all *Salmonella* serovars isolated from human cases [5]; it has been frequently isolated from contaminated vegetables, broiler, chicken, and the environments of slaughterhouses and layer farms worldwide [6]. This serovar is considered a relevant public health problem by the European Community together and was reported in 0.7% of human cases in 2009 and 2010 [7]. *S.* Virchow together with *S.* Typhimurium, *S.* Enteritidis, *S.* Hadar and *S.* Infantis has been included in the monitoring program as indicated in the zoonoses Directive 2003/99/CE as well as the control program for breeding, laying and broiler flocks of *Gallus gallus* (Regulation (EC) No 2160/2003). *S*. Virchow strains resistant both to amikacin and gentamicin, as well as to third generation cephalosporins [8] and with reduced susceptibility to fluoroquinolones [9], have been reported. The emergence of antimicrobial resistance is of particular concern because this serovar seems to show a predilection for extra-intestinal infection [9]. Moreover, whereas non-typhoid *Salmonella* bacteremia is usually associated with a favorable outcome in children it can be life-threatening in adults [10].

In Italy, between 1997 and 2013, only six pediatric cases of meningitis due to *Salmonella* infection were reported, and none of these were of Group C (data from National Surveillance Systems for invasive bacterial diseases, ISS, Rome, Italy, unpublished data) [11]; hence, to the best of our knowledge, we here describe the first case in Italy of *S.* Virchow meningitis in an adult. A previous study in the United Kingdom reported that *S.* Virchow caused eight cases of bacteremia in adults; although none of the patients developed meningitis, all were secondary to gastroenteritis and two had a travel history in Spain [12]. In addition, three cases of meningitis following *S.* Virchow food poisoning in adults were reported in the United Kingdom [13] and one case was documented in Scotland in an 18-year-old patient [14]. Furthermore, two cases of meningitis caused by *S.* Virchow have been described in Sweden and in Germany [4,15] which shared some features with the case in Italy in that both the patients travelled abroad (Thailand and Denmark, respectively), were not part of an outbreak and the source of infection remains unknown. However, for the Italian patient it is possible that the meningitis occurred after a foodborne infection 2 months prior to onset, and because antibiotic administration for other pathologies may have prolonged the intestinal carriage of the bacterium [16]. The fact that the patient received a prolonged course of both ciprofloxacin and levofloxacin is likely to have contributed to the reduced susceptibility to ciprofloxacin of the strain. In addition, according to laboratory data and anamnesis, immunodepression of the patient could not be excluded, although further clinical information was not available and he did not report any underlying medical conditions.

## Conclusions

This report further highlights the risk of meningitis as a presentation of salmonellosis and, to the best of our knowledge, describes the first case in Italy of *S.* Virchow meningitis in a young adult patient which was probably related to a long carriage state after a previous episode of gastroenteritis. Of interest, the infection was independently detected by the two Italian surveillance systems for invasive bacterial diseases and for human gastrointestinal infections (ENTER-NET Italia), providing additional evidence of the usefulness of dedicated surveillance systems. In fact, infection by *S.* Virchow is rare and the likely route of transmission remains unclear and requires further surveillance. Parry *et al*. [17] in a recent retrospective study showed that *S.* Virchow and Panama were more commonly associated with bacteremia and concluded that secondary bacteremia can be associated with an adverse outcome in non-human immunodeficiency virus (HIV)-infected adults admitted to hospital with non-typhoidal *Salmonella* gastroenteritis. Hence, antibiotic therapy can be considered in patients at high risk of secondary bacteremia, including elderly patients (>65 years) and younger adults with underlying chronic conditions.

## Consent

Written informed consent was obtained from the patient for publication of this case report and accompanying images. A copy of the written consent is available for review by the Editor-in-Chief of this journal.

## Competing interests

The authors declare that they have no competing interests.

## Authors’ contributions

DL, SM and AS analyzed and interpreted patient data from the medical chart and laboratory records and provided epidemiological information. CL performed the microbiological characterization of the strain and analyzed data from the literature. IL and GR designed the case report form, conducted the literature review and were the major contributors in writing the manuscript. All authors read and approved the final manuscript.
